# Supplementary material for: Reconstructing the incidence rate and immune fraction of the population via a single snapshot survey: A case study of COVID-19 in Japan
Source: PLoS Comput Biol. 2026 Mar 6;22(3):e1013990. doi: 10.1371/journal.pcbi.1013990 (PMC12991366; doi:10.1371/journal.pcbi.1013990)
Supplement: S1 Text — (PDF) [file pcbi.1013990.s001.pdf]

S1 Text: Survey items and the questionnaire used in this study

S1.1 Survey Items

1. Biological sex
2. Age
3. Diagnosis of COVID-19 in Feb 2024
4. Last month of infection (if any)
5. Last month of vaccination (if any)
6. Underlying health conditions
   -Diagnosed with diabetes (regardless of type 1 or type 2).
   -Currently untreated or under treatment for a malignant neoplasm.
   -Under treatment with immunosuppressive drugs, including adrenal corticosteroids, for conditions other than the above.
   -Currently or previously diagnosed with heart disease, such as myocardial infarction, angina, arrhythmia, or heart failure.
   -Currently diagnosed with one of the following: bronchial asthma, chronic obstructive pulmonary disease (COPD), interstitial pneumonia, pulmonary embolism, pulmonary hypertension, or bronchiectasis (excluding asthma limited to childhood).
   -Currently under treatment for or diagnosed with cerebrovascular diseases, such as cerebral infarction or cerebral hemorrhage.
   -Diagnosed with chronic liver diseases, such as liver cirrhosis, fatty liver, alcoholic liver disease, or autoimmune hepatitis.
   -Body Mass Index (BMI) of 30 kg/m^2 or higher: BMI = weight (kg) / [height (m) × height (m)].
7. Drinks alcohol at least once a week on average (regardless of the amount).
8. Smoking habit (including occasional smoking, not necessarily daily).
9. Household size (1 or >1)
10. Prefecture of residence
11. Job categories: based on the 2020 Census (1,2)
    -Agriculture, Forestry
    -Fisheries
    -Mining, Quarrying, and Gravel Extraction
    -Construction
    -Manufacturing
    -Electricity, Gas, Heat Supply, and Water Utilities
    -Information and Communications

-Transportation and Postal Services
-Wholesale and Retail Trade
-Finance and Insurance
-Real Estate and Goods Rental and Leasing
-Academic Research, Professional, and Technical Services
-Accommodation and Food Services
-Living-Related and Amusement Services
-Education and Learning Support
-Medical and Welfare Services
-Combined Services
-Services (not elsewhere classified)
-Public Administration (not elsewhere classified)
-Industry Not Classifiable
-Unemployed

S1.2: Questionnaire (English-translated version)

Definitions: In this survey, "infection with COVID-19" is defined as meeting at least one of the following two criteria, regardless of the presence or absence of symptoms.

1. **A positive result from a PCR test. The testing location (e.g., hospital, public health center, testing center) and sample type (e.g., saliva, nasal swab) are not specified.**
2. **A positive result from an antigen test (test kit).**

Q1. Please indicate your biological sex.

- **Male**
- **Female**

Q2. Please enter your age.

( ) years old

(Validation: Integer from 0 to 100)

Q3. Were you diagnosed with COVID-19 in February 2024?

- **Yes**
- **No**

Q4. When were you last diagnosed with COVID-19 between February 2020 and January 2024?

- **None**
- **(Dropdown list of months from February 2020 to January 2024)**

Q5. When did you last receive a COVID-19 vaccination?

- **None**
- **(Dropdown list of months from February 2021 to January 2024)**

Q6. Please select all applicable underlying health conditions from the list below.

- **Diagnosed with diabetes (regardless of type 1 or type 2).**
- **Currently untreated or under treatment for a malignant neoplasm (so-called 'cancer').**
- **Under treatment with immunosuppressive drugs, including adrenal corticosteroids, for conditions other than the above.**
- **Currently or previously diagnosed with heart disease, such as myocardial infarction, angina, arrhythmia, or heart failure.**
- **Currently diagnosed with one of the following: bronchial asthma, chronic obstructive pulmonary disease (COPD), interstitial pneumonia, pulmonary embolism, pulmonary hypertension, or bronchiectasis (excluding asthma limited to childhood).**
- **Currently under treatment for or diagnosed with cerebrovascular diseases, such as cerebral infarction or cerebral hemorrhage.**
- **Diagnosed with chronic liver diseases, such as liver cirrhosis, fatty liver, alcoholic liver disease, or**

autoimmune hepatitis.

- **Body Mass Index (BMI) of 30 kg/m² or higher: BMI = weight (kg) / [height (m) × height (m)].**
- **None of the above.**

Q7. Please select all applicable lifestyle habits from the list below.

- **Drinks alcohol at least once a week on average (regardless of the amount).**
- **Smoking habit (including occasional smoking, not necessarily daily).**
- **None of the above.**

Q8. Please indicate your current prefecture of residence.

Prefecture (   )

e.g., Kyoto Prefecture, Tokyo Prefecture, Okinawa Prefecture

Q9. Please indicate your household size.

- **1 person**
- **2 or more persons**

Q10. Please indicate your job category.

- **Agriculture, Forestry**
- **Fisheries**
- **Mining, Quarrying, and Gravel Extraction**
- **Construction**
- **Manufacturing**
- **Electricity, Gas, Heat Supply, and Water Utilities**
- **Information and Communications**
- **Transportation and Postal Services**
- **Wholesale and Retail Trade**
- **Finance and Insurance**
- **Real Estate and Goods Rental and Leasing**
- **Academic Research, Professional, and Technical Services**
- **Accommodation and Food Services**
- **Living-Related and Amusement Services**
- **Education and Learning Support**
- **Medical and Welfare Services**
- **Combined Services**
- **Services (not elsewhere classified)**
- **Public Administration (not elsewhere classified)**
- **Industry Not Classifiable**
- **Unemployed / Not applicable**

S1.3: Questionnaire (original in Japanese)

○定義事項

　今回のアンケートにおいて、「新型コロナウイルス感染症への感染」は以下の 2 つのうち少なくとも 1 つを満たす場合とします。症状の有無は問いません。

1. PCR 検査により陽性と判明した場合。検査場所（病院、保健所、検査所等）および検体の種類（唾液、鼻腔ぬぐい液等）は問いません。
2. 抗原検査（検査キット）により陽性と判明した場合。

＜基本情報＞

設問1　あなたの性別（生物学的性）を教えてください。

○　男性

○　女性

設問2　あなたの年齢を教えてください。

（　　　）歳

（入力値 validation：0~100 までの整数）

設問3　2024 年２月に新型コロナウイルス感染症と診断されましたか？

○はい

○いいえ

設問4　2020 年 2 月～2024 年 1 月の間で、最後に新型コロナウイルス感染症と診断された時期を教えてください

（「なし」、または▼2020 年 2 月～2024 年 1 月の年月リスト）

設問5　最後に新型コロナウイルスのワクチン接種を受けたのはいつですか？

（▼2021 年 2 月～2024 年 1 月の年月リスト）

設問6　基礎疾患として、以下の中で該当があるものを教えてください。

○　糖尿病(1 型、2 型を問わない)と診断されたことがある

○　悪性新生物(いわゆる「がん」)で、未治療または治療中の状態である

○　上記以外で、副腎皮質ステロイド薬をはじめとする免疫抑制薬で治療中である

○　心筋梗塞、狭心症、不整脈、心不全などの心疾患に罹患中または既往がある

○　気管支喘息、慢性閉そく性肺疾患（COPD)、間質性肺炎、肺塞栓症・肺高血圧症、気管支拡張症のいずれかに罹患中である(小児期のみの喘息は除く)

○　脳血管疾患（脳梗塞、脳出血など）の治療中、または診断されたことがある

○　肝硬変、脂肪肝、アルコール性肝障害、自己免疫性肝炎等の慢性肝疾患と診断されたことがある

○　BMI が 30kg/m^2 以上: BMI=体重(kg)/[身長(m)×身長(m)]

○　なし

設問7　生活習慣として、以下に該当があるものをすべて選択してください。

〇　平均して週に 1 回以上の飲酒(量は問わない)

〇　喫煙している（毎日でなくても、時々吸う日がある場合も含む)

〇　なし

設問8　あなたが現在お住まいの都道府県を教えてください。

都道府県（　　　　　　　　　　）

例：京都府、東京都、沖縄県

設問9　世帯の人数を教えてください

〇1 人

〇2 人以上

設問10　あなたの職業属性を教えてください。

〇　農業，林業

〇　漁業

〇　鉱業，採石業，砂利採取業

〇　建設業

〇　製造業

〇　電気・ガス・熱供給・水道業

〇　情報通信業

〇　運輸業，郵便業

〇　卸売業，小売業

〇　金融業，保険業

〇　不動産業，物品賃貸業

〇　学術研究，専門・技術サービス業

〇　宿泊業，飲食サービス業

〇　生活関連サービス業，娯楽業

〇　教育，学習支援業

〇　医療，福祉

〇　複合サービス事業

〇　サービス業（他に分類されるものを除く）

〇　公務（他に分類されるものを除く）

〇　分類不能の産業

Reference

1. Employment Security Bureau,Ministry of Health, Labour and Welfare. List of Industry Classification Codes, Major Categories (in Japanese) [Internet]. 2023 [cited 2024 Dec 11]. Available from: https://www.hellowork.mhlw.go.jp/info/industry_list01.html

2. Statistics Bureau、Ministry of Internal Affairs and Communications. 2020 Population Census (in Japanese) [Internet]. 2024 [cited 2024 Dec 11]. Available from: https://www.stat.go.jp/data/kokusei/2020/index.html
