# Supplementary material for: Reconstructing the incidence rate and immune fraction of the population via a single snapshot survey: A case study of COVID-19 in Japan
Source: PLoS Comput Biol. 2026 Mar 6;22(3):e1013990. doi: 10.1371/journal.pcbi.1013990 (PMC12991366; doi:10.1371/journal.pcbi.1013990)
Supplement: S2 Text — (PDF) [file pcbi.1013990.s002.pdf]

S2 Text: Prior distributions and information used for Bayesian inference by Markov Chain Monte Carlo method

1: Immune protection and its decay

$$v_E \sim Beta(10, 10),$$
$$E\!: all\ types\ of\ exposure. \tag{S1}$$

$$h^{\{1\}}\left(=\frac{\log(2)}{\gamma^{\{1\}}}\right) \sim Normal(60, 10), \tag{S2}$$

$$h^{\{2\}}\left(=\frac{\log(2)}{\gamma^{\{2\}}}\right) \sim Normal(360, 120), \tag{S3}$$

$$f_{infect}, f_{vaccine} \sim Beta(20, 20), \tag{S4}$$

For v_E and f, priors were arbitrarily provided by assuming mild concentrations around 50%. Priors for $h^{\{1\}}$ and $h^{\{2\}}$ were based on those used in Hogan et al (1) with minor modification.

2: Force of Infection

$$\beta_0 \sim Norma\ l(0, 10), \tag{S5}$$

$$\beta_{i\geq 1} \sim Laplace(0, \tau), \tag{S6}$$

$$\tau \sim Half\ Cauchy(0, 1), \tag{S7}$$

$$\delta_{pref_i} \sim Normal(0, \sigma_\delta), \tag{S8}$$

$$\sigma_\delta \sim Inverse\ Gamma(2, 0.5), \tag{S9}$$

The prior distribution for β_0 was arbitrarily set, whereas Laplace distributions as priors for $\beta_{i\geq 1}$ were designed to be defined by hyperparameter τ. Prefectural effects δ_{pref_i} were assumed to follow normal distributions with standard deviation σ_δ, that is also a hyperparameter. Note that a constraint $\sum_i \delta_{pref_i} = 1$ was posed to ensure identifiability of δ_{pref_i}.

3: Weighting

$$\boldsymbol{\alpha} \sim Dirichlet(\boldsymbol{u}), \tag{S10}$$

$$\boldsymbol{u} = (5, 5, \dots, 5). \tag{S11}$$

All elements of $\boldsymbol{u}$ (a vector with 7166 elements) were arbitrarily set to 5, leading to the assumption that each value in $\boldsymbol{\alpha}$ has approximately 1% probability of taking values less than $\frac{1}{4\times 7166}$, or one-fourth of the expected probability without additional information.

4. Published studies on the protection against JN.1 subvariant infection by vaccine or infection

Using the protection function $R(\Delta\tau)$ in analogy to $V_i(t)$ in equation (1) in the main text:

$$V(\Delta\tau|E) = v_E\left[f_E * \exp\left(-\gamma^{\{1\}}\Delta\tau\right) + (1 - f_E) * \exp\left(-\gamma^{\{2\}}\Delta\tau\right)\right], \quad \text{(S12)}$$

we defined the following likelihoods based on published studies:

a) Kirwan et al. (2)

$$\log\left(1 - V(30|vaccine, XBB)\right) \sim Normal\left(\log(1 - 0.422), \frac{log(1 - 0.217) - log(1 - 0.603)}{3.92}\right), \quad \text{(S13)}$$

$$\log\left(1 - V(90|vaccine, XBB)\right) \sim Normal\left(log(1 - 0.241), \frac{log(1 - 0.007) - log(1 - 0.429)}{3.92}\right), \quad \text{(S14)}$$

$$\log\left(1 - V(150|vaccine, XBB)\right) \sim Normal\left(log(1 - 0.267), \frac{log(1 + 0.275) - log(1 - 0.579)}{3.92}\right), \quad \text{(S15)}$$

$$\log\left(1 - V(30|vaccine, Wuhan + Omicron\ BA.1/5)\right) \sim Normal\left(log(1 - 0.022), \frac{log(1 + 0.357) - log(1 - 0.295)}{3.92}\right), \quad \text{(S16)}$$

$$\log\left(1 - V(90|vaccine, Wuhan + Omicron\ BA.1/5)\right) \sim Normal\left(log(1 - 0.151), \frac{log(1 + 0.554) - log(1 - 0.536)}{3.92}\right), \quad \text{(S17)}$$

$$\log\left(1 - V(90|infection, \quad XBB)\right) \sim Normal\left(log(1 - 0.493), \frac{log(1 - 0.292) - log(1 - 0.636)}{3.92}\right). \quad \text{(S18)}$$

b) Huiberts et al.(3)

$$\log\left(1 - V(30|vaccine, XBB)\right) \sim Normallog(1 - 0.45), \frac{\left(log(1 - 0.3) - log(1 - 0.6)\right)}{3.92}. \quad \text{(S19)}$$

c) Link-Gelles et al.(4)

$$\log\left(1 - \frac{1 - V(80|vaccine, XBB)}{1 - V(674|vaccine, Wuhan + Omicron\ BA.1/5)}\right) \sim Normal\left(log(1 - 0.49), \frac{log(1 - 0.19) - log(1 - 0.68)}{3.92}\right). \qquad (S20)$$

Reference

1. Hogan AB, Doohan P, Wu SL, Mesa DO, Toor J, Watson OJ, et al. Estimating long-term vaccine effectiveness against SARS-CoV-2 variants: a model-based approach. Nat Commun. 2023 Jul 19;14(1):1–10.

2. Kirwan PD, Foulkes S, Munro K, Sparkes D, Singh J, Henry A, et al. Protection of vaccine boosters and prior infection against mild/asymptomatic and moderate COVID-19 infection in the UK SIREN healthcare worker cohort: October 2023 to March 2024. J Infect. 2024 Sep 27;89(5):106293.

3. Huiberts AJ, Hoeve CE, de Gier B, Cremer J, van der Veer B, de Melker HE, et al. Effectiveness of Omicron XBB.1.5 vaccine against infection with SARS-CoV-2 Omicron XBB and JN.1 variants, prospective cohort study, the Netherlands, October 2023 to January 2024. Euro Surveill. 2024 Mar 7;29(10):2400109.

4. Link-Gelles R, Ciesla AA, Mak J, Miller JD, Silk BJ, Lambrou AS, et al. Early estimates of updated 2023-2024 (monovalent XBB.1.5) COVID-19 vaccine effectiveness against symptomatic SARS-CoV-2 infection attributable to co-circulating omicron variants among immunocompetent adults - Increasing Community Access to testing program, United States, September 2023-January 2024. MMWR Morb Mortal Wkly Rep. 2024 Feb 1;73(4):77–83.
