# Supplementary material for: Reconstructing the incidence rate and immune fraction of the population via a single snapshot survey: A case study of COVID-19 in Japan
Source: PLoS Comput Biol. 2026 Mar 6;22(3):e1013990. doi: 10.1371/journal.pcbi.1013990 (PMC12991366; doi:10.1371/journal.pcbi.1013990)
Supplement: S1 Table — (PDF) [file pcbi.1013990.s003.pdf]

S1 Table. Descriptive analysis of survey respondents

Category	Total	COVID-19 in Feb 2024	No COVID-19 In Feb 2024	% diagnosed In Feb 2024
Total	7166	251	6915	3.53
Age Group (years)				
20-29	1196	79	1117	6.61
30-39	1187	66	1121	5.56
40-49	1197	41	1156	3.43
50-59	1190	28	1162	2.35
60-60	1194	18	1176	1.51
70 and over	1202	19	1183	1.58
Sex				
Male	3592	145	3447	4.04
Female	3574	106	3468	2.97
History of Vaccination				
No	1257	20	1237	1.59
Yes	5678	231	5678	4.07
Last Vaccination				
XBB.1.5	1373	38	1335	2.77
Wuhan + Omicron BA. 1/5	2060	56	2004	2.72
Wuhan	2476	137	2339	5.53
Infection before Dec 2023				
No	4867	49	4818	1.01
Yes	2299	202	2097	8.79
Diabetes Mellitus	343	25	318	7.29
Neoplastic Disorder	109	25	84	22.94
Immune Suppression	91	16	75	17.58
Respiratory Disorder	219	27	192	12.33
Cardiovascular Disorder	220	21	199	9.55
Cerebrovascular Disorder	91	10	81	10.99
Liver Disorder	100	12	88	12
Obesity (BMI >30kg/m^2)	240	10	230	4.17
Smoking	866	26	840	3
Drinking	1293	50	1243	3.87

Household size				
1	1529	60	1469	3.92
More than 1	5637	191	5446	3.39
