# Supplementary material for: Reconstructing the incidence rate and immune fraction of the population via a single snapshot survey: A case study of COVID-19 in Japan
Source: PLoS Comput Biol. 2026 Mar 6;22(3):e1013990. doi: 10.1371/journal.pcbi.1013990 (PMC12991366; doi:10.1371/journal.pcbi.1013990)
Supplement: S2 Table — (PDF) [file pcbi.1013990.s004.pdf]

S2 Table. Number of respondents by job category linked to the Census

Job Category	
Total	7166
Agriculture, Forestry	72
Fisheries	6
Mining, Quarrying, and Gravel Extraction	15
Construction	231
Manufacturing	768
Electricity, Gas, Heat Supply, and Water Utilities	59
Information and Communications	217
Transportation and Postal Services	295
Wholesale and Retail Trade	504
Finance and Insurance	174
Real Estate and Goods Rental and Leasing	125
Academic Research, Professional, and Technical Services	91
Accommodation and Food Services	183
Living-Related and Amusement Services	114
Education and Learning Support	292
Medical and Welfare Services	605
Combined Services	55
Services (not elsewhere classified)	483
Public Administration (not elsewhere classified)	270
Industry Not Classifiable	214
Unemployed	2393
