# Supplementary material for: Reconstructing the incidence rate and immune fraction of the population via a single snapshot survey: A case study of COVID-19 in Japan
Source: PLoS Comput Biol. 2026 Mar 6;22(3):e1013990. doi: 10.1371/journal.pcbi.1013990 (PMC12991366; doi:10.1371/journal.pcbi.1013990)
Supplement: S3 Table — (PDF) [file pcbi.1013990.s005.pdf]

S3 Table. Estimated values of parameters characterizing immune protection dynamics

Parameters	Estimated values (95% CrI)
$v_{infect,XBB}$	0.555 (0.380, 0.712)
$v_{infect,pre-XBB\ Omicron}$	0.611 (0.392, 0.797)
$v_{infect,pre-Omicron}$	0.499 (0.290, 0.705)
$v_{vaccine,XBB}$	0.548 (0.452, 0.650)
$v_{vaccine,Wuhan+\ Omicron\ BA.1/5}$	0.347 (0.213, 0.495)
$v_{vaccine,Wuhan}$	0.441 (0.239, 0.661)
$h^{\{1\}}\ \left(=\frac{\log(2)}{\gamma^{\{1\}}}\right)$ (days)	62.0 (41.7, 81.5)
$h^{\{2\}}\ \left(=\frac{\log(2)}{\gamma^{\{2\}}}\right)$ (days)	439.5 (253.1, 644.3)
f_{infect}	0.412 (0.273, 0.570)
$f_{vaccine}$	0.524 (0.372, 0.669)
