# Supplementary material for: Reconstructing the incidence rate and immune fraction of the population via a single snapshot survey: A case study of COVID-19 in Japan
Source: PLoS Comput Biol. 2026 Mar 6;22(3):e1013990. doi: 10.1371/journal.pcbi.1013990 (PMC12991366; doi:10.1371/journal.pcbi.1013990)
Supplement: S4 Table — [26,27]. (PDF) [file pcbi.1013990.s006.pdf]

S4 Table. Comparison of our national incidence estimates with the case count estimates provided by the Moderna Inc. website (URL: https://moderna-epi-report.jp/) in February 2024.

Age Group	Moderna Estimates (in thousands)	Estimates, present study (in thousands)
20-59	1187	2018 (1884, 2163)
60 or over	432	759 (652, 872)
