# Supplementary material for: Reconstructing the incidence rate and immune fraction of the population via a single snapshot survey: A case study of COVID-19 in Japan
Source: PLoS Comput Biol. 2026 Mar 6;22(3):e1013990. doi: 10.1371/journal.pcbi.1013990 (PMC12991366; doi:10.1371/journal.pcbi.1013990)
Supplement: S5 Table — (PDF) [file pcbi.1013990.s007.pdf]

S5 Table. Comparison of the proportion of key personal-level attributes between those in the “lower-FoI (FoI<0.045)” mode and “higher-FoI(FoI>=0.045)” for those aged 20-29, weighted results as national estimates

Category	Higher FoI (% with 95% CrI)	Lower FoI (% with 95% CrI I)
Female ratio	48.3 (46.2, 50.1)	50.0 (48.6, 51.6)
Last Vaccination		
None	15.5 (13.8, 17.3)	26.4 (24.7, 28.2)
XBB.1.5	4.6 (3.8, 5.6)	6.8 (5.7, 8.2)
Wuhan + Omicron BA. 1/5	24.4 (22.6, 26.5)	25.3 (23.4, 27.3)
Wuhan	55.4 (53.2, 57.6)	41.4 (39.3, 43.6)
Last Infection before Dec 2023		
None	0.8 (0.0, 3.0)	99.9 (96.8, 100)
XBB sublineages	28.4 (26.3, 30.5)	0.0 (0.0, 1.2)
Pre-XBB Omicron	42.6 (40.4, 44.9)	0.0 (0.0, 1.6)
Pre-Omicron	28.0 (26.0, 30.1)	0.0 (0.0, 0.6)
No Exposure at all	0.1 (0.0, 1.1)	26.4 (24.6, 28.1)
Diabetes Mellitus	1.4 (0.9, 2.6)	1.4 (0.7, 1.9)
Neoplastic Disorder	1.7 (1.3, 2.3)	0.0 (0.0, 0.0)
Immune Suppression	2.6 (2.0, 3.3)	0.3 (0.0, 0.7)
Respiratory Disorder	6.1 (4.8, 7.6)	0.8 (0.0, 1.7)
Cardiovascular Disorder	2.5 (1.9, 3.4)	0.2 (0.0, 0.4)
Cerebrovascular Disorder	1.5 (0.9, 2.4)	0.0 (0.0, 0.0)
Liver Disorder	0.8 (0.6, 1.1)	0.0 (0.0, 0.1)
Obesity (BMI $>30kg/m^2$)	1.7 (0.8, 2.3)	2.1 (1.4, 3.0)
Smoking	5.9 (4.7, 7.0)	4.7 (3.8, 5.8)
Drinking	8.5 (7.4, 9.8)	6.3 (5.3, 7.6)
Household size >1	72.1 (69.7, 74.3)	67.0 (65.0, 69.0)
Infection History	99.2 (97.0, 100)	0.1 (0.0, 3.26)
