# Supplementary material for: Reconstructing the incidence rate and immune fraction of the population via a single snapshot survey: A case study of COVID-19 in Japan
Source: PLoS Comput Biol. 2026 Mar 6;22(3):e1013990. doi: 10.1371/journal.pcbi.1013990 (PMC12991366; doi:10.1371/journal.pcbi.1013990)
Supplement: Attachment — Submitted filename: okada_rebuttal_v2.docx [file pcbi.1013990.s028.docx]

[Point-by-point responses to Reviewers] Reconstructing the force of infection and immune fraction of the population via a single snapshot survey: a case study of COVID-19 in Japan (submitted to PLOS Computational Biology)

[Response to Reviewer 1]

We appreciate your thorough and very helpful review. Please find below the point-by-point response to your comment.

<Overall>

1) In this paper, the authors report estimates of COVID-19 case incidence, strain-specific susceptibility, and vaccine effectiveness from a snapshot online questionnaire in Japan. The work yields interesting epidemiological results and proposes a novel methodological approach. I can see many people in public health being interested in this work as an inexpensive way to generate insights that have until now only been possible with very expensive studies.

I did find the innovation in the paper difficult to access. There were very few details of the survey questions, and the key methodological innovation was not described early on in natural language. I think PLOS Computational Biology still allows a "methods first" structure. If so, I'd suggest adding a concise natural language methods section to explain what was asked in the questionnaire and how the Bayesian framework works (not a load of equations—natural language about what the equations are doing). The current methods with equations could then be included in the supplementary material.

I think this is very good and important work, but it was hard to tell from a single careful reading, and I feel very familiar with the questions and the methods.

>>

Response:

Thank you very much for your kind comments with very substantial advice.

Firstly, we followed the “methods first” structure in the revised manuscript. The revised methods section includes a more thorough explanation of the details of the questionnaire, and how our model works.

(However, because we believe that the methodological details should also be provided in equations, we tried to balance both the descriptive and equation-based explanations.)

By both the restructuring and further revision, we clarified the key takeaways and the impact of our study. However, we also welcome further advice on this point if necessary.

<Detailed>

2) Abstract – should give a hint of how this can be achieved without any biological outcome data.

>>

Response:

We added a description to clearly state that our questionnaire survey collected information that are used in conventional vaccine effectiveness studies. (L24-25)

3) Results – it's still very much a mystery at this point how we can infer these quantities from a single snapshot survey. A "results first" ordering might not make sense for this work.

>>

Response:

We reorganized to “methods first” structure accordingly so that readers may more clearly understand how we inferred the estimates as described in the Results section.

4) Page 13, line +3 (pdf numbering): need to define and motivate the β (beta) parameter. Very abrupt for the reader as written.

>>

Response:

We added a description on the beta (intercept and coefficients) as the parameters that are first described in Eq. 3 of the Method section. (L 275-L276)

5) Page 16, +2: the reweighting seems to be more of a methods detail, or perhaps the first results?

>>

Response:

The structure we assumed is

-1: Describe results that do not necessarily translate into population-level estimates (result sections 3.1 and 3.2 in our revised manuscript).

-2: Show results from the “weighting” that are needed for “translation” into population-level estimates

-3: Show “translated” results with interpretations.

In this sense, we still consider that this ordered structure should be maintained, because the validity of our “weighting” can only be confirmed by showing tables in section 3.3. However, given your comment, we realized that our intention and context were not very clearly expressed. We added a sentence at the beginning of section 3.4. “COVID-19 incidence by age group in February 2024” for clarification. (L351-352)

We believe the “methods first” restructuring may also help the readability regarding this issue.

6) Page 18, -4: what time period do the results relate to? How wide was the snapshot?

>>

Response:

We clarified the time period and the width in L351-352

7) Page 24, line 1: These findings should be presented as part of the results, not only highlighted from the discussion. How big were the differences? Are they material to the other claims in the paper?

>>

Response:

On the gap between our estimates and serosurveys, we added a summary to the Results section (L363-373) and also provided full details in S4 Fig, S5 Fig, and S2 Data. Any infection was mostly 20–40% lower for <60y and ~10–25% lower for ≥70y; any exposure was ~10–20% lower overall. The overall tendency is that our model estimates of the rates of “any infection” and “any exposure” are consistently lower than those suggested by two serological datasets.

In addition to the serological survey, we also added comparative analysis on the national incidence in February 2024 with the estimated national incidence of COVID-19 in Japan provided by a website managed by Moderna Inc. (<https://moderna-epi-report.jp/>). This comparison revealed that our estimate was higher by around 50-100% for both age 20-59 and age 60 or over (S4 table)

Given that serosurveys and Moderna estimates are methodologically distinct and based on different datasets or underlying samples, we believe our estimates fall within the plausible range, which also suggests that the statistically weighted estimates overall in our study are reasonable.

We added relevant discussion on this issue. (L489- L495)

8) Page 24, -8: no details at this point about the monthly time scales.

>>

Response:

We should have clarified the time scale in the methods section, and made due corrections in the methods section (L 152)

9) Page 29, +3: vaccine effectiveness against self-reported infection? The details of the outcome measure need to be stated more clearly, especially given that the questionnaire was in Japanese and the article is in English. What were participants actually asked? The details of the questionnaire are not clear. Given recent LLM advances in translation, perhaps the authors could provide the original Japanese text and one or two LLM-generated translations to give readers a better chance to understand the outcome.

>>

Response:

The questionnaire was basically designed to collect information on the diagnosis of COVID-19 regardless of symptoms. However, in Japan, no efforts such as contact tracing or screening programs that aim to capture potentially asymptomatic cases were underway since 2022. Thus, we considered “diagnosis” as a proxy for “symptomatic infection”. We clarified these backgrounds in L143-145.

We should have provided the original questionnaire for the readers to clarify what we asked the respondents. We added the questionnaire (both the original Japanese version and its English translation) to the S1 text.

We also agree that the definition of “infection” throughout the text was not emphasized enough, so we added a relevant description in the Methods section (L139-145)

10) Page 30, +1: This appears to be the central methodological innovation. The questionnaire is designed to inform a multi-group attack-rate calculation. So people's risk of infection in a similar group in a similar place should be consistent with the rest of their group. This does make sense – can we know how good a fit the data are to the model? This structure should be adding considerably more constraint than a simple regression approach—so does it look like it's working?

>>

Response:

We should have provided posterior predictive checks (PPCs) for “whether or not infected in February 2024”. To address your point, we added

-S2 Fig: PPC figure by sex in each decadal age group

-S1 Data: PPC table by prefecture

to the supplementary materials. We also added relevant descriptions in L272-275 in the revised manuscript.

[Response to Reviewer 2]

We truly appreciate your very fundamental advice on our manuscript. Please find below the point-by-point response to your comment.

<Overall>

1) This article investigates the estimation of COVID-19 incidence and protection conferred by previous infections and vaccinations using a single cross-sectional Internet-based survey conducted in Japan in February 2024. The authors propose a statistical framework to reconstruct the force of infection (FoI) and individual-level protection based on reported immunization history and health-related covariates. While the study is methodologically interesting, I have major concerns regarding several points:

>>

Response:

Thank you very much for your detailed advice. Please check the following responses and the renewed manuscript to make sure that due revisions were made. We welcome additional comments from you if necessary.

<Detailed>

2) The study would benefit significantly from validation against objective external data sources, such as case counts, serological survey, hospitalisation data. While the authors mention some comparison with sero-surveys in the supplementary materials, this is limited and not well integrated into the main results. Such validation is crucial for confirming the accuracy of incidence and protection estimates derived from self-reported data.

>>

Response:

Given that there are limited data sources that are available for comparison of national estimates, we conducted an additional comparison of our results with those provided by a website managed by Moderna Inc. (<https://moderna-epi-report.jp/>). We compared our national result with that from the Moderna website and added the results for both age 20-59 and age 60 or over (S4 table). Our estimate was higher by 50-100% compared with their point estimates overall.

Together with the comparison between the nationwide serological survey, we added a summary on the comparison with external data in the Results section (L363-L373)

Combining the abovementioned findings together, our estimates are likely to fall into the plausible range among various approaches to estimate population-wide incidence or history of infection. An additional discussion on this issue was added in the Discussion section. (L489-L495)

We also realized that the nature of “self-reporting” was not clearly described in the original text. We added descriptions to the methods section (L139-145) and also added the questionnaire text used in our study in the latter part of S1 text.

3) It is unclear what it means to model and report protection at one-month post-immunization (Figure 1), when many immunizing events—particularly natural infections or vaccinations—occurred well before the study period, even more than two years before for the pre-Omicron infections. Although the protection curve is modeled (or rather, depicted) over six months post-event, the actual data used for fitting includes participants with much older immunization histories. This creates a disconnect between the temporal assumptions of the model and the actual time lags in the data, raising concerns about too much extrapolation. A clarification would be welcome.

>>

Response:

The underlying assumption in our immunity waning model is that the biological process governing the “fast-decay” and “slow-decay”, and the fraction of these, are shared among the types of last exposure considered in our study. This follows prior work by Hogan et al (https://pubmed.ncbi.nlm.nih.gov/37468463/), which builds on immunological observations described in Khoury et al. (https://pubmed.ncbi.nlm.nih.gov/34002089/), and our model can be considered as a simplified version of Hogan et al.

Given these assumptions, besides the shared decay parameters and the fraction of fast and slow waning protection, the parameter that needs to be estimated independently by exposure types is the peak protection level. Although this parameter may essentially read as protection at around 1-month post-immunization, it serves as an anchor that captures the waning process that is not explained by the shared parameters. Accordingly, our model is not designed to extrapolate beyond the unobserved data but rather to describe overall waning dynamics.

However, we realized that the current manuscript does not comprehensively convey the abovementioned discussion. We further clarified this point in the methods section. (L203-204, L215-217)

4) Related to the previous point I think the authors should provide either a figure or table showing raw or minimally processed data, such as:

- Type of last immunizing event (infection or vaccination),

- Time since the event,

- Whether the respondent was infected in February 2024.

>>

Response:

We agree that this should be described as basic information. We added a new descriptive figure (S1 Fig) and a relevant mention in the methods section. (L156)

5) Many individuals report last immunization months (or years) prior to the survey, yet no reinfection was observed between that time and February 2024. Would it be beneficial to the study to use a survival analysis or time-to-event model here? This non-infection interval contains valuable information that is currently unused, such as the protection given by immunization not only at the time of the survey but also during all opportunities of infections in the meantime.

>>

Response:

We did not collect information on all the “infection” and “vaccine” events up to February 2024, so we do not have complete information on both the outcomes and the key modifiers of infection risk. This makes it difficult to conduct survival analysis or time-to-event models with a comprehensive dataset.

However, we truly appreciate your comment that sheds light on possible future directions beyond the present work. We added relevant discussion (L499-502)

We realized that the underlying information on the nature of our questionnaire is not fully stated regarding this issue, so details on the questionnaire itself were added to the latter part of S1 text.

6) The bimodal distribution of FoI in younger individuals (Figure 3) is interesting but should be explored more thoroughly. The authors should clarify what immunization types or demographics are associated with each mode.

>>

Response:

We regret an error in the code used to produce Figs. 3–5 that is now corrected. This error stemmed from rounding, which was causing unintended undercounting of the lowest FoI or Protection fraction. Due to corrections on the bin definitions, the low-FoI patterns now appear slightly differently than in the original figures. The MCMC was not rerun, so these corrections do not essentially affect the study’s conclusions.

We divided the respondents aged 20-29 by FoI threshold of 0.045 by visual inspection. The resulting comparison of the weighted proportion of personal attributes including covariates in the model, together with key vaccine and infection history, is added as a table in the supplementary materials (S5 table)

These revealed that the rates of previous vaccination or infection, several comorbidities were higher in the higher-FoI group. However, the “never-infected” rate was higher in the lower-FoI group, despite the lower vaccination rate.

We also added relevant text describing the findings from this additional exploration. (L463-465)

7) In relation to Figure 5, the authors mention a subgroup in the “low force of infection – low protection” region. If possible, could the authors clarify whether these individuals are a specific group of unexposed (e.g., never infected, low contact) or whether their presence is simply a statistical expectation of the model?

>>

Response:

To clarify this issue, we decomposed the heat map in the main text (Fig 5) by the type of last immunizing event, that is shown as a newly added figure. (S6 Fig) It can be seen that the never-exposed population almost strictly falls in the low FoI- low (no) Protection area. However, this figure also reveals that a substantial proportion of low FoI-low Protection consists of those whose last immunizing event was vaccination, and very few contributions to this area were made by those whose last immunizing event was infection. In summary, the low protection-low FoI density consisted of both the never-exposed group and the “last event = vaccine” group.

Thus, the low FoI- low (no) Protection consists of both the never-exposed population and part of the statistical expectations of the vaccinated population, across all age groups. We added relevant descriptions to the Result section. (L414-418)

Our questionnaire did not explicitly collect information that may serve as a direct proxy for contact rates, but it is possible that more cautious contact patterns are seen among some unvaccinated populations. We added a relevant discussion on this issue. (L463-465)
