# Supplementary material for: Reconstructing the incidence rate and immune fraction of the population via a single snapshot survey: A case study of COVID-19 in Japan
Source: PLoS Comput Biol. 2026 Mar 6;22(3):e1013990. doi: 10.1371/journal.pcbi.1013990 (PMC12991366; doi:10.1371/journal.pcbi.1013990)
Supplement: Attachment — Submitted filename: okada_rebuttal_rev2.docx [file pcbi.1013990.s029.docx]

[Point-by-point responses to Reviewers] Reconstructing the incidence rate and immune fraction of the population via a single snapshot survey: a case study of COVID-19 in Japan (formerly: Reconstructing the force of infection and immune fraction of the population via a single snapshot survey: a case study of COVID-19 in Japan)

(submitted to PLOS Computational Biology)

[Response to Academic Editor]

We appreciate your thorough and very helpful review. The “We should have…”s in our previous responses were meant as you suggested. We apologize for the confusion.

[Response to Reviewer 2]

We truly appreciate your very fundamental advice on our manuscript. Please find below the point-by-point response to your comment.

1) I appreciated the efforts of the authors in responding to the questions of the reviewers, but I still have concerns regarding some results and the claims made in the study.

For instance, In really appreciated that they included external studies. However think more efforts should be made to discuss the discrepancy between their study and the external study (Moderna). If available, I think this paper would benefit from comparing the risk factors from those other studies. If seroprevalence estimates are different, are the trends in risk factors still reliable ?

>>

Response:

We agree that we should include further discussion on the discrepancy between external study and ours.

Regarding individual-level risk factors, both external studies (Moderna and seroprevalence) do not provide any information on the individual-level risk factors that underly their estimates. Therefore, validation of our estimates on the effect of individual-level risk factors cannot be conducted in the present study.

To clarify the abovementioned issues and emphasize the importance of validating our results in future studies, we revised the method and discussion section. (L194-196, L490-494)

2) I’m not convinced that increasing the size of the study as it is proposed by the authors in line 518-519 would benefit so much for survey-based estimate of the inference. Given the design of the study, the biases are essentially similar to that of case-based estimates of the incidence. I suggest the authors outline some potential sources of bias of their study. The first that come to my mind are asymptomatic infections that may lead to underestimating the incidence, and the second is a high incidence in the household, family members, colleagues that may increase the respondent probability of reporting symptoms, and third, high incidence within the population.

>>

Response:

We realized that our message in the relevant section was unclear and potentially confusing regarding the term “respondent bias”. Here “respondent bias” here was meant to describe that increasing the sample size would reduce the representativeness of our respondents for each census stratum. For clarity we revised the relevant sentence. (L495-497)

We also agree that the biases the reviewer listed is very important; we emphasized these biases in the revised manuscript. . (L475-L482)

3) There are other issues with the asymptomatic infections. Vaccination could induce more asymptomatic and paucisymptomatic infections (see for instance Joseph et al., JAMA, 2022 and many other studies). Those could be detected with serological surveillance but the proposed approach would fail to identify them. This doesn’t mean the approach is not useful, as it may still be able to identify and quantify risk factors for severe infections. In this sense it is an interesting approach, that can be implemented quickly. However, I suggest that the authors tone down their claim that they can assess the force of infection as they claim in the title. The authors themselves outline this as a limitation on line 150 where they say that they detect « symptomatic COVID-19 infections ».

>>

Response:

We agree that our claim regarding estimating “Force of Infection” might be misleading. Throughout the manuscript we made amendments referring to “diagnosis-based FoI” as suggested. (Title, L28, L32, L42, L51, L221-L222, L236, L364, L377, L414, L427)
